# Description of a New Cryptic Rhabditid, *Parasitorhabditis paraterebrana* n. sp. (Nematoda: Rhabditidae), with Remarks on Two Known Species from Korea

**DOI:** 10.2478/jofnem-2025-0027

**Published:** 2025-08-31

**Authors:** Abraham Okki Mwamula, Chang-Hwan Bae, Yi Seul Kim, Dong Woon Lee

**Affiliations:** Research Institute of Invertebrate Vector, Kyungpook National University, Sangju 37224, Republic of Korea; Biodiversity Research Department, Species Diversity Research Division, National Institute of Biological Resources, Incheon, 22689, Republic of Korea; Department of Entomology, Kyungpook National University, Sangju, 37224, Republic of Korea

**Keywords:** DNA barcodes, morphology, morphometrics, phylogeny, taxonomy

## Abstract

A new cryptic species of the genus *Parasitorhabditis* isolated from the bark of a dead pine tree was characterized using morphological features, morphometrics, and DNA barcodes. *Parasitorhabditis paraterebrana* n. sp. is characterized by its stoma 20–24 μm in depth; tips of prorhabdions not bent inwards; metarhabdions with two subventral, and two subdorsal teeth; corpus longer than postcorpus; hemizonid 15.0–26.5 μm posterior to excretory pore; vulva-anus distance 21.5–31.5 μm, ca equal to or slightly less than vulval body diameter; rectum distinctly longer than anal body diameter; female tail cupola-shaped, conoid posteriorly, with an extended spike; male with slender spicules, nearly straight to minimally curved towards a nearly acute to a bluntly rounded tip; and bursa with 10 pairs of bursal rays, with a 2 + 3 + 2 + 3 typical pattern. It differs from the morphologically similar *P. terebrana* by the non-bent tips of prorhabdions, the corpus being longer than postcorpus, the bursal rays’ pattern, and a more cupola-shaped tail in female and DNA barcodes. The DNA phylogenies using the 18S rRNA, 28S rRNA and *COI* gene markers showed well-supported sister relations of *Parasitorhabditis paraterebrana* n. sp. with *P. terebrana* and *P. obtusa*.

The genus *Parasitorhabditis* Fuchs, 1937 belongs to the family Rhabditidae *Örley*, 1880. The species of this genus are generally known to form parasitic or phoretic relationships with bark beetles, especially with members of the subfamily Scolytinae Latreille, and some members of the family Cerambycidae Latreille, 1802 (Poinar, 1975). Within the subfamily Scolytinae, *Parasitorhabditis* species are mainly associated with beetles of the genera *Dendroctonus* Erichson, 1836; *Ips* De Geer, 1775; *Scolytus* Geoffroy, 1762; *Dryocoetes* Eichhoff, 1864; *Hylurgops* LeConte, 1876; *Crypturgus* Erichson, 1836; *Hylastes* Erichson, 1836; *Hylurgus* Latreille, 1807; *Pityogenes* Bedel, 1888; *Cryphalus* Erichson, 1836; *Phloeosinus* Chapuis, 1869; and *Polygraphus* Erichson, 1836 (Massey, 1974; Sudhaus, 1974; Tomalak et al., 1989). Most of these species generally colonize the insect digestive tract, and their free-living forms inhabit the frass in the beetle galleries and bark of dead trees inhabited by the beetles (Grucmanová and Holuša, 2013; Mwamula et al., 2023).

The diagnostics of species of this genus is rather confounded. Descriptions of the majority of species were published between 1915 and 1980, and some of the published literature contain insufficient morphometric data for comprehensive comparative purposes according to the current demands of the discipline. Important morphometric characters such as stoma length, spicules length, ratio of corpus (pharyngeal procorpus + median bulb) to postcorpus (pharyngeal isthmus + basal bulb) length, definitive bursal rays’ number and pattern and De Man ratios were not supplied in some of the original species descriptions. The modern species identification approach of integrated taxonomy that combines detailed morphometric data and molecular phylogenetic studies can provide accurate species identification and delimitation within the genus. During a nematological survey conducted in 2024 in natural pine forest ecosystems in Korea, a population morphologically similar to *Parasitorhabditis terebrana* Massey, 1974 was discovered. Also, two other known species of the genus were recovered from the bark of dead black- and red pine (*Pinus thunbergii* Parl and *Pinus densiflora* for. *erecta*) tree stands. The detailed studies revealed that the former population represents a new cryptic species, designated as *Parasitorhabditis paraterebrana* n. sp. herein, and was described based on both morphological and molecular phylogenetic criteria. Additional morphological and molecular data of the two known species; *P. terebrana* and *P. obtusa* (Fuchs, 1915) Chitwood and Chitwood, 1950 are also supplied.

## Materials and Methods

### Nematode populations and extraction

Samples of the bark of dead pine-tree stands were taken from pine-tree forests in Uljin, and Gumi, Gyeongsangbuk-do Province, Republic of Korea. The nematode populations were extracted from the bark layer cuttings using the Baermann funnel method (Baermann, 1917). Nematode specimens belonging to *Parasitorhabditis* were handpicked from the collected nematode suspension under a Nikon SMZ1000 stereomicroscope (Nikon). Specimens were subsequently characterized based on inferences from morphometric and molecular data.

### Morphological characterization

The specimens were heat-killed, fixed with formaline-glycerine, and transferred to pure glycerin according to Seinhorst (1959) as modified by De Grisse (1969). The processed nematode specimens were mounted on permanent slides and observed under a light microscope. Morphometric data and photomicrographs were taken using a Zeiss imager Z2 microscope (Carl Zeiss) fitted with Axiovision, Material Science Software for Research and Engineering (Carl Zeiss). Line drawings were made using a drawing tube and redrawn using CorelDRAW® software version 24. Species identification and diagnosis were done following the diagnostic species compendia presented by Rühm (1956), Andrássy (1983), and Carta et al. (2010).

### Molecular characterization

Morphometrically confirmed, heat-relaxed female and male specimens were used in DNA extraction. Genomic DNA was extracted from the specimens using the method of Iwahori et al. (2000), with modifications. Briefly, nematodes were washed in drops of distilled water on a glass slide. A single cleaned nematode specimen was transferred into a 1-*μ*l drop of distilled water on a sterilized glass slide and crushed with a small filter paper chip (1 mm × 1 mm) with the aid of a forceps under a stereoscope microscope. The paper chip was subsequently suspended in a 30 μl nematode DNA lysis buffer containing proteinase K and incubated at 65 °C for 30 mins and 95 °C for 10 mins. The resultant solution was used as the DNA template for a polymerase chain reaction (PCR). PCR was performed using WizPure™ Taq DNA Polymerase kit in accordance with the manufacturer’s instructions. Four gene fragments (the nearly full-length 18S rRNA gene, the D2-D3 expansion segment of 28S rRNA gene, the partial ITS rRNA gene and the partial *COI* gene) were amplified and sequenced in this study. The 18S rRNA gene was amplified as two partially overlapping fragments using two primer sets: 988F (5′-CTCAAAGATTAAGCCATGC-3′) and 1912R (5′-TTTACGGTCAGAACTAGGG-3′), 1813F (5′-CTGCGTGAGAGGTGAAAT-3′) and 2646R (5′-GCTACCTTGTTACGACTTTT-3′) (Holterman et al., 2006); the primer pairs D2Ab (5′-ACAAGTACCGTGAGGGAAAGTTG-3′) and D3B (5′-TCGGAAGGAACCAGCTACTA-3′) (De ley et al., 1999) were used to amplify the D2-D3 expansion segment of 28S rRNA; COI-F1 (5′-CCTACTATGATTGGTGGTTTTGGTAATTG-3′) and COI-R2 (5′-GTAGCAGCAGTAAAATAAGCACG-3′) (Kanzaki and Futai, 2002) were used to amplify the partial *COI* gene; TW81 (5′-GTTTCCGTAGGTGAACCTGC-3′) and AB28 (5′-ATATGCTTAAGTTCAGCGG GT-3′) (Curran et al., 1994) were used to amplify the partial ITS rRNA gene of *P. terebrana* and *P. paraterebrana* n. sp.; and S_ITS1 (5′-TTGATTACGTCCCTGCCCTTT-3′) and Vrain2R (5′-TTTCACTCGCCGTTACTAAGGGAATC-3′) (Vrain et al., 1992) were used to amplify the partial ITS rRNA gene of *P. obtusa.* PCR was executed with a thermal cycler model T100™, Bio-Rad. The thermal cycling program using the primer sets 988F/1912R, 1813F/2646R, D2Ab/D3B, TW81/AB28, and COI-F1/COI-R2 were as described by Mwamula et al. (2023), and the thermal profile using S_ITS1/Vrain2R primer set was as detailed by Mwamula et al. (2024). Purification of the PCR products was performed using the QIAquick PCR Purification Kit (Qiagen) and quantification was done using a quickdrop spectrophotometer (Molecular Devices). The purified PCR products were directly sequenced with the primers specified above at Macrogen Inc. The edited new sequences were submitted to the NCBI GenBank database under the accession numbers: PQ589232-PQ589236 (for 18S rRNA); PQ589220-PQ589225 (for 28S rRNA); PQ589226-PQ589231 (for ITS rRNA), and PQ585733-PQ585737 (for the *COI* gene).

### Phylogenetic analyses

The new sequences (18S rRNA, D2-D3, ITS rRNA and *COI* gene) were compared with those of related species of *Parasitorhabditis* using the BLAST homology search program. Multiple alignments for sequences of the genus, including comparable sequences of related species from other genera published in GenBank (Kanzaki et al., 2013, 2015, 2016; Valizadeh et al., 2017; Bhat et al., 2020; Mwamula et al., 2023) were built using ClustalX (Thompson et al., 1997). The sequences of *Rhabditoides inermiformis* (Osche, 1952) Dougherty, 1955 (AF083017) and *Rhomborhabditis regina* (Schulte and Poinar, 1991) Sudhaus, 2011 (AF082997) were selected as the outgroup sequences for the 18S rRNA gene; sequences of *Rhabditoides inermiformis* (EF990727) and *Rhomborhabditis regina* (EF990726) were selected as outgroups for 28S rRNA gene; and those of *Chylorhabditis epuraeae* Kanzaki, Hamaguchi, and Takeuchi-Kaneko, 2021 (LC601016) and *Rhabditis* Dujardin, 1844 species (OL468734) were the outgroups for *COI* gene. The ITS rRNA gene phylogeny was not reconstructed. This is because, except for only four sequences of *P. terebrana*, there were no other definitive ITS rRNA gene sequences of *Parasitorhabditis* or other related genera in GenBank database for comparison. Bayesian inference (BI) of the three genes was performed using MrBayes 3.2.7 (Ronquist et al., 2012), with GTR + I + G model selected for all datasets. Bayesian analysis for each gene was initiated with a random starting tree and run with four chains for 1 × 10^6^ generations. The Markov chains were sampled at intervals of 100 generations. After discarding burn-in samples, consensus trees were generated with the 50% majority rule. The generated trees were visualized and edited using FigTree v1.4.4 software. Posterior probabilities (PP) exceeding 50% are given on appropriate clades. Intraspecific and interspecific sequence variations were analyzed using PAUP* v4.0a169 (Swofford, 2003).

## Results

**Figure 1:**
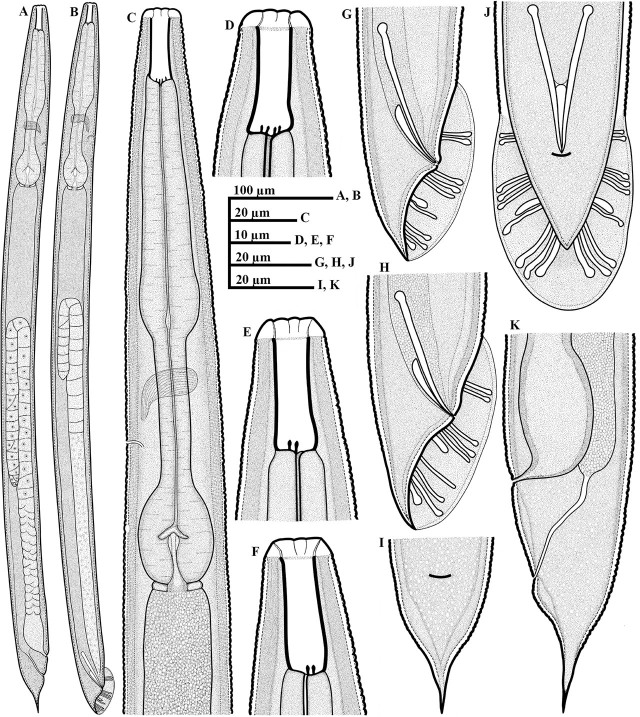
Line drawings of *Parasitorhabditis paraterebrana* n. sp. (A-K): A: Female whole body; B: Male whole body; C: Female anterior region, including the pharynx region; D, E, F: Variation in lip shape and arrangement of teeth (D: Lateral view with all the four teeth; E: The two subventral teeth; F: The two subdorsal teeth); G, H: Male tail in right lateral view, with arrangement of bursal rays; I: Female tail in ventral view; J: Male tail in ventral view, with arrangement of bursal rays; K: Female tail in lateral view.

**Figure 2:**
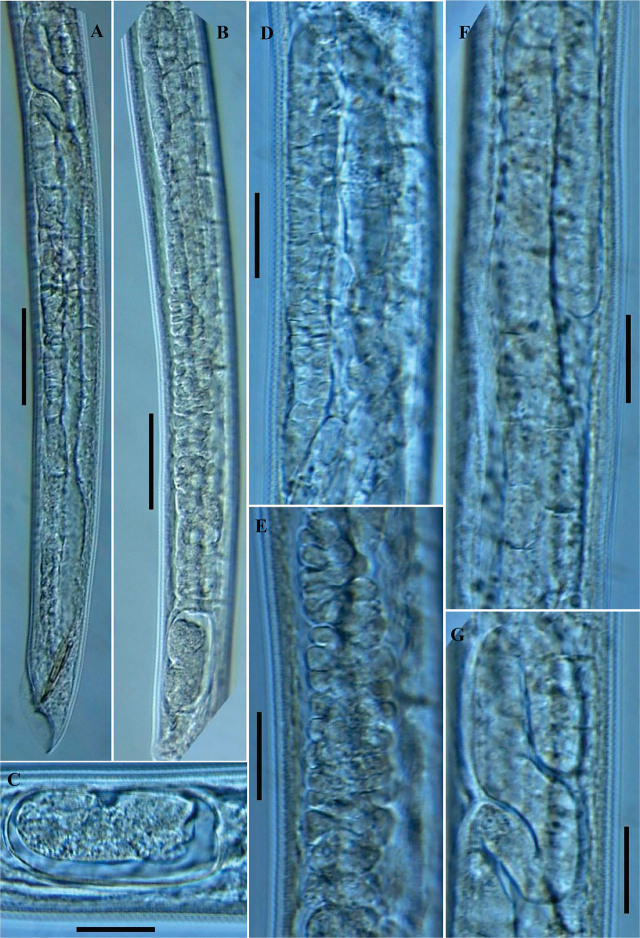
Photomicrographs of *Parasitorhabditis paraterebrana* n. sp. (A-G): A: Male reproductive system; B: Female reproductive system; C: Developing egg in the uterus; D: The reflexed region of ovary; E: The oviduct; F, G: The reflexed portion of the testis (Scale bars: A, B = 50 μm; C, D, E, F, G = 20 μm).

**Figure 3:**
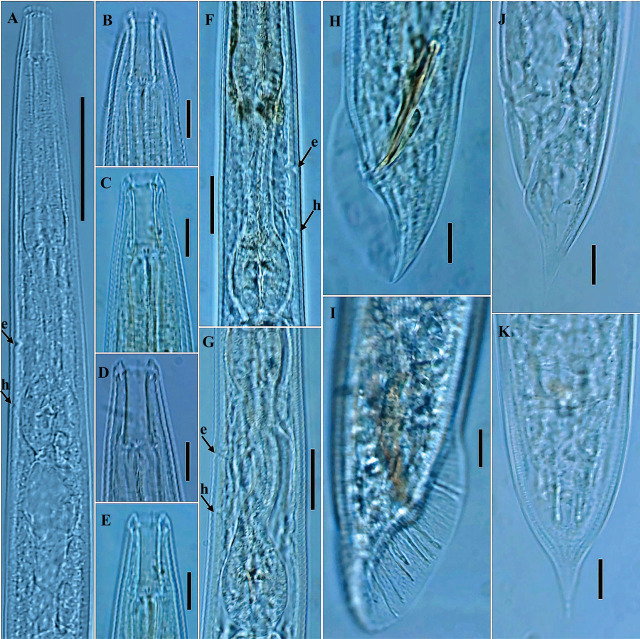
Photomicrographs of *Parasitorhabditis paraterebrana* n. sp. (A-K): A: Female anterior region, including the pharynx region (arrow e and h indicate the position of secretory-excretory pore and hemizonid, respectively); B, C, D, E: Variation in lip shape and arrangement of teeth (B: Lateral view with all the four teeth; C: The two subventral teeth; D, E: The two subdorsal teeth); F, G: Posterior part of pharynx (arrow e and h indicate the position of secretory-excretory pore and hemizonid, respectively); H, I: Male tail region; J: Female tail in lateral view; K: Female tail in ventral view. (Scale bars: A = 50 μm; B, C, D, E, H, I, J, K = 10 μm; F, G = 20 μm).

*Parasitorhabditis paraterebrana* n. sp. (Figs. 1–3).

### Measurements

See Table 1.

**Table 1:** Comparison of morphometrics of *Parasitorhabditis paraterebrana* n. sp. and *Parasitorhabditis terebrana* Massey, 1974 from Korea and topotype population by Massey (1974).

**Character**	***Parasitorhabditis paraterebrana* n. sp.**			***Parasitorhabditis terebrana***			

	**Holotype**	**Paratypes**		**Korea (Current study)**		**USA (Massey, 1974)**	

	♀	♀♀	♂♂	♀♀	♂♂	♀♀	♂♂
n	–	20	22	6	5	?	?
L	693.0	673.3±54.6 (598.0–814.0)	625.6±53.4 (553.5–732.5)	877.8±87.0 (780.5–995.0)	847.9±87.9 (746.0–979.0)	770–810	750
a	19.0	19.0±0.6 (17.9–20.0)	19.0±1.0 (16.9–21.7)	21.4±1.2 (20.1–23.7)	21.0±1.4 (19.0–22.5)	19.7–20.1	19.5
b	3.9	3.7±0.3 (3.2–4.3)	3.6±0.2 (3.3–4.2)	4.5±0.4 (4.0–5.1)	4.4±0.2 (4.1–4.6)	4.2–4.3	4.1
c	21.8	23.0±3.0 (18.3–28.6)	24.0±2.4 (20.8–28.8)	25.9±2.5 (20.7–28.3)	26.7±1.2 (25.2–28.4)	26.2–27.3	27.3
c’	1.6	1.7±0.3 (1.3–2.3)	1.5±0.1 (1.3–1.7)	1.6±0.2 (1.4–1.9)	1.4±0.2 (1.2–1.7)	–	–
V	91.6	91.7±0.7 (90.3–92.6)	–	92.9±0.6 (92.0–93.5)	–	93	–
G1/T (without flexure)	46.5	47.9±2.5 (44.5–53.2)	46.2±2.6 (41.0–51.4)	–	–	–	–
Lip height	4.0	4.3±0.4 (3.5–5.0)	3.7±0.5 (3.0–4.5)	3.3±0.1 (3.0–3.5)	3.1±0.2 (3.0–3.5)	–	–
Lip diameter	11.0	11.3±0.7 (10.0–12.5)	10.8±0.7 (9.5–12.5)	12.5±0.4 (12.0–13.0)	11.7±0.4 (11.0–12.5)	–	–
Stoma length	22.0	21.9±1.2 (20.0–24.0)	21.4±1.2 (19.5–23.5)	20.5±0.7 (19.5–21.5)	20.0±1.5 (18.0–22.0)	21	–
Stoma width	5.5	5.2±0.4 (4.5–6.0)	4.2±0.4 (3.5–5.0)	5.0±0.4 (4.0–5.5)	4.1±0.4 (3.5–5.0)	–	–
Anterior to median valve	89.0	92.3±3.8 (83.5–99.0)	87.1±4.2 (77.0–92.5)	100.5±5.8 (93.5–108.0)	98.9±3.4 (93.5–102.5)	–	–
Anterior to nerve ring	116.5	112.2±7.6 (98.5–123.0)	101.5±6.9 (89.5–114.0)	110±7.4 (102.5–122.0)	–	–	–
Excretory pore	126.0	128.1±7.5 (113.0–141.0)	123.9±6.2 (112.5–139.0)	120.2±6.0 (100.5–125.0)	–	–	–
Pharynx length	176.0	179.6±6.5 (167.0–190.0)	173.3±7.9 (159.0–187.0)	193.5±9.6 (184.0–209.0)	193.4±13.1 (180.0–212.0)	–	–
Maximum body diameter	36.5	35.4±2.8 (31.0–43.0)	33.0±3.4 (27.0–40.0)	41.0±2.7 (38.0–44.5)	40.4±2.5 (38.0–45.0)	–	–
Vulval body diameter	29.5	28.0±2.4 (24.5–34.5)	–	34.1±2.4 (30.5–38.5)	–	–	–
Vulva to anus length	25.0	24.6±2.2 (21.5–31.5)	–	30.7±2.6 (28.0–35.0)	–	–	–
Vulva to tail tip	57.0	54.3±4.6 (46.5–65.0)	–	64.8±5.2 (57.0–70.5)	–	–	–
Anal / cloacal body diameter	20.0	17.7±1.5 (14.0–20.5)	17.9±1.2 (15.5–20.0)	20.8±0.9 (19.5–22.0)	22.2±2.1 (20.0–25.0)	–	–
Tail length	32.0	29.7±3.9 (22.5–37.0)	26.1±2.2 (22.5–30.0)	34.1±3.9 (28.5–39.0)	31.6±2.0 (29.0–34.5)	–	–
Spicules	–	–	34.1±2.0 (30.5–37.0)	–	36.4±1.4 (34.5–38.0)	–	34
Gubernaculum	–	–	15.3±0.9 (14.0–17.0)	–	16.2±0.6 (15.5–17.0)	–	17

All measurements are in μm and in the form mean ± standard deviation (range).

### Description

#### Female

General habitus straight or slightly arcuate when heat-killed and fixed. Cuticle 2.0–2.5 μm thick at mid-body, moderate to prominently annulated. Lips angular to rounded, 3.5–5.0 μm high and 10.0–12.5 μm wide, with visible pseudolip sectors and moderately prominent papillae. Stoma 20.0–24.0 μm in depth and 4.5–6.0 μm wide, with its walls slightly broader at base. Anterior tips of prorhabdions not bent inwards. Remnants of metarhabdions with two subventral teeth, and two subdorsal teeth, visible in lateral view. Pharynx muscular throughout, without clear median bulb demarcation. Lumen of corpus prominently sclerotized with transverse ridging. Procorpus including the moderately swollen base ca 1.1–1.3 times the postcorpus length. Nerve ring at mid- or anterior to mid-isthmus, 98.5–123.0 μm from anterior end. Excretory pore posterior to, or rarely at level of nerve ring, 113.0–141.0 μm from anterior end. Hemizonid visible, located 15.0–26.5 μm posterior to excretory pore or 135.0–164.5 μm from anterior end. Lateral field hardly discernible. Reproductive system monodelphic-prodelphic, arranged from anterior (distal) as ovary, oviduct, spermathecal-uterus junction, crustaformeria, uterus, vagina/vulva, extending 44.5–53.2% of total body length. Distal (anterior) part of ovary reflexed dorsally, extending beyond ovary-oviduct junction in some specimens or covering 36–78% of gonad system length. Oocytes arranged in two rows in the reflexed part of ovary, and well-developed oocytes arranged in a single row near oviduct. Oviduct comprised by small ellipsoidal cells, not conspicuously distinct from anterior ovary in some specimens, connected to an enlarged wider posterior part (spermatheca) comprised by large, oval cells, forming an indistinct roundish to oblong-shaped sac. Spermathecal-uterus junction distinct, connected by a band of thread-like tissue, surrounded by oval cells. Uterus length varying according to presence or absence of embryo, composed of irregularly arranged globular uterine cells, forming prominent crustaformeria in the distal part, and the wider proximal tubular part with epithelial wall. Uterus often containing 1–2 developing eggs, 54–57 μm long and 24–26 μm wide. Vagina short, oblique with muscular walls. Lips of vulva not protuberate. Vulva a transverse slit. Vulva-anus distance 21.5–31.5 *μ*m, ca equal to or slightly less than vulval body diameter (0.8–1.0 times), also ca equal or less than tail length (0.6–1.1 times). Rectum distinctly longer than anal body diameter. Phasmid indistinct under light microscope. Tail cupola-shaped, conoid posteriorly, with an extended moderate spike.

#### Male

Generally as abundant as females. General morphology similar to that of female except for sexual characters and conoid tail with an acute terminus. Lips angular to rounded, 3.0–4.5 μm high and 9.5–12.5 μm wide, with moderately prominent papillae. Stoma as in female in depth. Procorpus including the moderately swollen base ca 1.1–1.4 times the postcorpus length. Nerve ring anterior to mid-isthmus, 89.5–114.0 μm from anterior end. Excretory pore and hemizonid as in female. Testis single, on right subventral region of intestine, outstretched, extending 41–51% of total body length, anteriorly reflexed ventrally or dorsally up to 20–51% of its length. An extended double flexure in germinal zone observed in some specimens. Spermatocytes arranged in two to three rows in reflexed part, and mainly two rows in the middle part. *Vas deferens* tube-like, occupying ca 23–26% of total gonad length, composed of large cells, joining the rectum and forming a narrow cloacal tube terminating into the cloaca. Spicules slender, nearly straight to minimally curved towards a nearly acute to a bluntly rounded tip. Gubernaculum slipper-shaped, posteriorly (away from tip) thickened, ca half or less than spicules length (0.4–0.5 times). Tail conoid with an acute terminus. Bursa peloderan with 10 pairs of bursal rays (typical of the genus). The second, eighth and ninth pairs directed dorsally, and others directed ventrally, with 2 + 3 + 2 + 3 or 2 + 3 + (2 + 3) typical ray pattern (Figs. 1G, 1J, and 3I), occasionally appearing in a 2 + 4 + (1+3) pattern (Fig. 1H). In lateral view, the first two pairs located precloacally, and almost reaching the edge of the bursa or ending behind it. Postcloacally, three or four pairs positioned close together, located just after cloacal opening, ending just behind the bursal edge (the three anterior pairs). The sixth bursal pair appears tube-like, much shorter, thicker, and stouter than all others. Seventh, eighth, and ninth rays end just slightly behind the bursal edge, with the ninth appearing a little longer than the two others. The last (10^th^) pair also appear shortened and thicker than the preceding pairs.

### Diagnosis and relationships

*Parasitorhabditis paraterebrana* n. sp. is characterized by having a medium sized body 0.55–0.81 mm long in both sexes; stoma 20.0–24.0 μm in depth; anterior tips of prorhabdions not bent inwards; remnants of metarhabdions with two subventral teeth, and two subdorsal teeth; corpus longer than postcorpus; hemizonid visible, 15.0–26.5 μm posterior to excretory pore; vulva-anus distance of 21.5–31.5 μm, ca equal to or slightly less than vulval body diameter, also ca equal or less than tail length; rectum distinctly longer than anal body diameter; female tail cupola-shaped, conoid posteriorly, with an extended moderate spike; spicules slender, nearly straight to minimally curved towards a nearly acute to a bluntly rounded tip; and bursa with 10 pairs of bursal rays with a 2 + 3 + 2 + 3 or 2 + 3 + (2 + 3) typical ray pattern, occasionally appearing in a 2 + 4 + (1+3) pattern.

*Parasitorhabditis paraterebrana* n. sp. looks very similar to *P. terebrana* in morphology, having two visible subventral teeth, and two subdorsal teeth and sharing almost all morphometric values. However, it differs from *P. terebrana* by having non-bent anterior tips of prorhabdions vs. anterior tips of prorhabdions bent inwards (see Massey (1974) and Mwamula et al. (2023)), a corpus being longer than the postcorpus vs. being equal to or slightly shorter than the postcorpus; the rectum being distinctly longer than anal body diameter vs. being almost equal; bursal rays in the pattern 2 + 3 + 2 + 3 or 2 + 3 + (2 + 3), occasionally appearing as 2 + 4 + (1+3) vs. 2 + (3+1) + 4; a more cupola-shaped tail in females; and differences in their DNA barcodes (details provided below). Based on morphometrics, *Parasitorhabditis paraterebrana* n. sp. is also close to *P. crypturgophila* Rühm, 1956, *P. opaci* Rühm, 1956, *P. palliati* (Fuchs, 1937) Skrjabin, Shikhobalova, Sobolev, Paramonov, and Sudarikov, 1954, *P. ateri* (Fuchs, 1915) Dougherty, 1955, *P. autographi* (Fuchs, 1937) Skrjabin, Shikhobalova, Sobolev, Paramonov, and Sudarikov, 1954 and *P. frontali* Carta, Bauchan, Hsu, and Yuceer, 2010. It differs from the *P. crypturgophila* by its longer stoma (20–24 vs. 16–19 μm), the presence of four teeth vs. no teeth, a corpus longer than the postcorpus vs. equal in length to postcorpus, longer gubernaculum (14–17 vs. 9–14 μm), and shorter vulva to anus distance (21.5–31.5 vs. 32–35 μm); from *P. opaci* by the longer stoma (20–24 vs. 17–19 μm), the presence of four teeth vs. three teeth, a cupola-shaped tail with an extended spike vs. conical tail; a lower b ratio (3.2–4.3 vs. 4.4–5.2); a shorter vulva to anus distance (21.5–31.5 vs. 30–35 *μm*), *and bursal ray*s in the pattern 2 + 3 + 2 + 3 or 2 + 3 + (2 + 3), occasionally appearing as 2 + 4 + (1+3) vs. 2 + 3 + 5. The new species differs from *P. palliati* by its shorter spicules (30.5–37.0 vs. 39–44 μm), a shorter gubernaculum (14–17 vs. 18–23 μm), bursal rays in the pattern 2 + 3 + 2 + 3 or 2 + 3 + (2 + 3), occasionally appearing as 2 + 4 + (1+3) vs. 2 + 4 + 4, the presence of four teeth vs. three teeth, a shorter vulva to anus distance (21.5–31.5 vs. 32–35 μm), a lower b ratio (3.2–4.3 vs. 5–6), and a cupola-shaped tail with an extended spike vs. conical tail. The new species differs from *P. ateri* by having shorter spicules (30.5–37.0 vs. 39–42 μm), a shorter gubernaculum (14–17 vs. 18–21 μm), the presence of four teeth vs. two-three teeth, bursal rays in the pattern 2 + 3 + 2 + 3 or 2 + 3 + (2 + 3), occasionally appearing as 2 + 4 + (1+3) vs. 2 + 4 + 4, a shorter vulva to anus distance (21.5–31.5 vs. 32–35 μm), a lower b ratio (3.2–4.3 vs. 4.9–6.6), and a cupola-shaped tail with an extended spike vs. conical tail. The new species differs from *P. autographi* by the presence of four vs. two teeth, lower b and c ratios (3.2–4.3 vs. 4.8–5.3 and 18.3–28.6 vs. 29–59, respectively), a shorter vulva to anus distance (21.5–31.5 vs. 35 μm), a longer pharynx (167–190 vs. 155–161 μm), the position of the vulva (V = 90.3–92.6 vs. 92–95), a cupola-shaped tail with an extended spike vs. a conical tail; and bursal rays in the pattern a 2 + 3 + 2 + 3 or 2 + 3 + (2 + 3), occasionally appearing as 2 + 4 + (1+3) vs. 2 + 5 + 3. The new species differs from *P. frontali* by the presence of four teeth vs. one tooth, a corpus longer than postcorpus vs. shorter than postcorpus, a longer pharynx (167–190 vs. 113–136 μm), a longer stoma (20–24 vs. 13–17 μm); and a longer gubernaculum (14–17 vs. 11.1–12.8 μm).

### Type habitat and locality

The species was recovered from the bark of a dead red pine (*Pinus densiflora* for. *erecta*) tree from Geumgang pine-tree forest in Uljin, Gyeongsangbuk-do Province. GPS coordinates: 37°02′84″N, 129°27′51″E.

### Type material

The holotype female, twelve female paratypes, and fifteen male paratypes were deposited in the National Institute of Biological Resources of Korea and eight female and seven male paratypes were deposited in the Nematode Collection of Kyungpook National University (KNU), Republic of Korea.

### Etymology

The species epithet refers to the Greek preposition *para*, “alongside of,” resembling the morphologically closest species, *Parasitorhabditis terebrana*.

Korean population of *Parasitorhabditis terebrana*

### Measurements

See Table 1.

### Remarks

The morphological and morphometric data of the studied population agree well with those of the paratype specimens studied by Massey (1974), and the subsequent details given by Mwamula et al. (2023), except for the relatively long body in the latter population (780.5–995 vs. 922–1244 *μm*). Also, nine bursal rays were recorded in the original description by Massey (1974). However, 10 bursal rays are evident in the currently studied population, with the ray pattern similar to the descriptions given by Mwamula et al. (2023). This observation is also in agreement with Andrássy (1983) and Sudhaus and Fitch (2001), who noted that the number of bursal rays (papillae) is constant in the genus *Parasitorhabditis*, i.e., 10 pairs (two of the ten pairs preanal). According to Massey (1974), the diagnostic characters of the species include the corpus being equal in length to isthmus and basal bulb, the unique tip of the prorhabdions bent inwards and the tail terminus shape. These features are seen in the population recovered and studied here, and the DNA sequences are identical to the previously published sequences for *P. terebrana* (Mwamula et al., 2023). The reproductive system is generally similar to that of *Parasitorhabditis paraterebrana* n. sp., arranged from anterior (distal) as ovary, oviduct, spermathecal-uterus junction, crustaformeria, uterus, vagina/vulva, extending 46–57% of total body length. Distal part of ovary reflexed dorsally, covering 37–70% of the total gonad system length. Oocytes arranged in two to three rows in the reflexed part of ovary, and in a single row near oviduct. Oviduct comprised by small ellipsoidal cells, connected to an enlarged wider posterior part composed of large cells, forming a roundish to oblong-shaped sac. Spermathecal-uterus junction distinct, connected by a band of thread-like tissue. Uterus composed of irregularly arranged globular uterine cells, forming prominent crustaformeria, and the wider proximal tubular part with epithelial wall. The uterus often contains 1–3 developing eggs. Vagina short, oblique with muscular walls. Lips of vulva mostly protuberate. Vulva a transverse slit. Male with a single testis, on right subventral region of intestine, outstretched, extending 44–56% of total body length, anteriorly reflexed ventrally or dorsally up to 22–54% of its length. Spermatocytes arranged in two to three rows in reflexed part. Vas deferens tube-like, occupying ca 24–27% of total gonad length, composed of large cells, joining the rectum and forming a narrow cloacal tube terminating into the cloaca.

**Figure 4:**
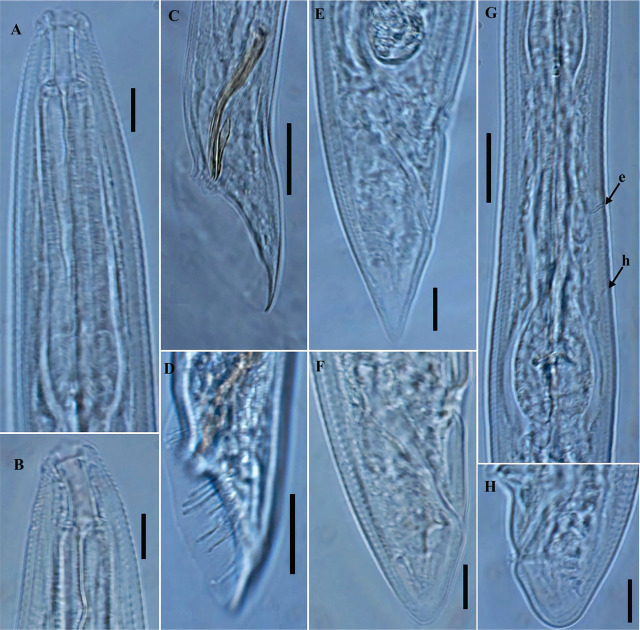
Photomicrographs of *Parasitorhabditis obtusa* (Fuchs, 1915) Chitwood and Chitwood, 1950 (A-H). A, B: Female anterior region; C, D: Male tail region; E, F, H: Variation in shape of female tail; G: Posterior part of pharynx (arrow e and h indicate the position of secretory-excretory pore and hemizonid, respectively). (A, B, E, F, H = 10 μm; C, D, G = 20 μm).

Korean population of *Parasitorhabditis obtusa* (Fig. 4)

### Measurements

See Table 2.

**Table 2:** Comparison of morphometrics of *Parasitorhabditis obtusa* (Fuchs, 1915) Chitwood & Chitwood, 1950 from Korea with topotype and other populations.

**Character**	**Korea (Current study)**		**Iran (Valizadeh et al., 2017)**		**Germany? (Rühm, 1956)**		**Germany (Fuchs, 1915)**	

	♀♀	♂♂	♀♀	♂♂	♀♀	♂♂	♀♀*	♂♂*
n	15	15	10	10	?	?	2	1
L	951.1±92.4 (784.0–1148)	753.9±66.3 (635.0–872.0)	959.0±92.0 (810–1067)	746±104 (624–925)	728–1115	751–855	957–1121	640
a	24.3±1.7 (21.7–27.0)	24.6±2.3 (20.9–28.4)	20.0±2.5 (16.9–23.6)	21.1±3.6 (14.4–27.2)	16.5–24.0	19.5–21.0	–	–
b	5.0±0.4 (4.4–6.0)	4.6±0.3 (4.0–5.0)	5.1±0.5 (4.5–5.6)	4.3±0.5 (3.6–5.3)	4.0–5.8	4.2–4.6	–	–
c	40.5±5.0 (34.2–49.9)	17.3±1.4 (14.8–19.6)	67.0±12.3 (50.0–88.9)	35.9±5.0 (28–46)	44.0–49.5	17.8–22.2	–	–
c’	1.2±0.2 (0.8–1.3)	2.0±0.2 (1.7–2.4)	0.7±0.1 (0.6–0.9)	1.4±0.3 (1.0–1.8)	–	–	–	–
V	94.5±0.6 (93.4–95.3)	–	95.8±0.9 (93.8–96.7)	–	94.0–95.7	–	–	–
G1/T (without flexure)	57.0±3.2 (49.9–62.7)	57.4±3.3 (52.6–64.4)	55.8±6.9 (41.5–63.5)	56.4±6.4 (47.5–65.6)	–	–	–	–
Lip height	4.4±0.4 (4.0–5.5)	3.5±0.3 (3.0–4.0)	–	–	–	–	–	–
Lip diameter	12.4±0.7 (11.5–13.5)	10.3±0.5 (9.5–11.5)	–	–	–	–	–	–
Stoma length	17.7±1.1 (15.5–19.5)	16.1±0.8 (15.0–17.5)	17.4±1.3 (16–20)	15.5±1.2 (13–17)	(16.0–20.0)	16.0–21.0	17.0	17.0
Stoma width	4.2±0.5 (3.5–5.5)	3.3±0.4 (2.5–4.0)	–	–	–	–	–	–
Anterior to median valve	82.6±3.5 (75.0–88.5)	72.8±2.9 (68.5–78.0)	–	–	–	–	–	–
Anterior to nerve ring	98.7±5.2 (91.5–108.0)	94.9±6.3 (84.5–111.5)	132.0±9.3 (121–150)	114.0±8.4 (100–127)	–	–	–	–
Excretory pore	124.3±4.2 (119.5–134.5)	115.1±6.7 (102.0–126.0)	143.0±8.2 (125–155)	126.0±7.6 (115–142)	–	–	–	–
Pharynx length	189.2±8.1 (169.0–201.0)	165.1±7.3 (150.5–173.0)	189.0±9.4 (172–201)	172.0±7.5 (156–183)	183.0–234.0	168.0–185.0	–	–
Maximum body diameter	39.1±3.0 (34.0–44.5)	30.7±2.0 (27.5–34.5)	48.1±4.3 (42–55)	35.9±5.0 (28–46)	42.0–53.0	39.0–44.0	–	34.0
Vulval body diameter	30.3±2.6 (26.5–35.5)	–	37.1±3.2 (33–42)	–	–	–	–	–
Vulva to anus length	23.1±3.4 (19.5–32.0)	–	–	–	21.0–43.0	–	34.0	–
Vulva to tail tip	46.8±5.1 (38.0–57.0)	–	–	–	–	–	54.0	–
Anal / cloacal body diameter	20.6±1.5 (18.0–25.0)	21.8±1.5 (19.0–23.5)	21.2±2.3 (18–26)	23.4±2.8 (20–28)	–	–	–	–
Tail length	23.7±3.0 (18.5–27.0)	43.7±2.4 (40.0–50.0)	14.6±2.7 (12–19)	31.6±7.2 (23–42)	18.0–25.0	38.0–46.0	20.0	–
Spicules	–	43.7±5.4 (33.0–49.0)	–	35.1±3.5 (31–42)	–	30.0–32.0	–	33.0
Gubernaculum	–	17.8±0.8 (16.0–19.0)	–	15.0 ± 1.6 (13–17)	–	15.0–18.0	–	14.0

All measurements are in μm and in the form mean ± standard deviation (range).

* *Parasitorhabditis obtusa* topotype population.

### Remarks

The morphology and morphometrics of the studied population agree well with those of the type population described by Fuchs (1915) and the subsequent morphometric data published by Rühm (1956), Massey (1956, 1960), Carta et al. (2010), and Valizadeh et al. (2017) except for the number of bursal rays reported by Massey (1960), with only 9 bursal rays illustrated in the line drawings, and Valizadeh et al. (2017), who reported 8–9 pairs of bursal rays although 10 pairs were illustrated in their line drawings (in lateral view). Additionally, the bursal rays arrangement pattern is not definitive in Valizadeh et al. (2017) (i.e., 4 rays in precloacal position and 6 rays post-cloacal). In the population studied here, a pattern of bursal rays as 2 + 3 + 2 + 3 was consistent and rarely appeared in a 2 + 3 + 3 + 2 pattern, agreeing with the illustrations of Rühm (1956). Additional characters reported in this study include corpus equal to postcorpus, excretory pore located posterior to nerve ring and 119.5–134.5 μm from anterior end. Hemizonid visible, located 24.0–32.5 μm posterior to excretory pore or 144.5–164.0 μm from anterior end, rectum distinctly longer than anal body diameter; gubernaculum lanceolate shaped, ending in an elongated acute tip (Fig. 4C). The line drawings of Rühm (1956) show that corpus is longer than postcorpus. This character was used in the compendium of the genus presented by Andrássy (1983), to distinguish *P. obtusa* from related species. However, in the original description by Fuchs (1915), the corpus was noted to be equal in length to the postcorpus, which also agrees with the line drawings of Valizadeh et al. (2017). Additional details on the reproductive system include the female reproductive system being generally arranged from the anterior (distal) as ovary, oviduct, uterus junction, crustaformeria, uterus, vagina/vulva, extending 53–64% of total body length. Distal part of ovary reflexed dorsally, covering 33–55% of the total gonad system length. Oocytes arranged in two to three rows in the reflexed part of ovary. Oviduct composed of small ellipsoidal cells anteriorly and enlarged posteriorly with large round cells. Spermathecal-uterus junction visible. Uterus composed of irregularly arranged globular uterine cells, forming prominent crustaformeria, and the wider proximal tubular part with epithelial wall. Uterus often contains 1–3 developing eggs. Vagina short, oblique with muscular walls. Lips of vulva protuberate or not protuberate. Vulva a transverse slit. Male with a single testis, on right subventral region of intestine, extending 53–64% of total body length, anteriorly reflexed dorsally up to 17–27% of its length. Spermatocytes arranged in two to three rows in reflexed part. Vas deferens tube-like, occupying ca 25–28% of total gonad length, composed of large cells, joining the rectum and forming a narrow cloacal tube terminating into the cloaca.

### Molecular characterization and phylogenetic relationships

The montaged two partially overlapping fragments of 18S rRNA gene sequences yielded ca 1700-bp long sequences for each studied species. No intraspecific sequence variation was recorded in the two newly obtained sequences (PQ589232, PQ589233) of *Parasitorhabditis paraterebrana* n. sp. In the 18S rRNA gene tree, sequences of *Parasitorhabditis paraterebrana* n. sp. were close to the few available sequences of other species of the genus. All sequences of *Parasitorhabditis* were grouped in a well-supported clade (PP=100). Notably, sequences of *Parasitorhabditis paraterebrana* n. sp. were highly similar to sequences of *P. terebrana* (OQ704209, OQ704209 and PQ589234), differing by only 4 bp (0.3%). *Parasitorhabditis paraterebrana* n. sp., sequences were also highly similar to the newly obtained sequences of *P. obtusa* (PQ589235, PQ589236), *P. obtusa* isolates accessioned EU003189, and KJ705089; and an unidentified *Parasitorhabditis* sp. (AF083028), differing by only 4–8 bp (0.4–0.5%). However, interspecific sequence differences of 31–33 bp (3.9–4.2%) were observed when compared with *P. obtusa* isolates accessioned MH590260 and the unidentified *Parasitorhabditis* spp. sequences (MH590259 and MH590261) from Taiwan.

The new 18S rRNA gene sequence for *P. terebrana* (PQ589234) was identical to *P. terebrana* sequences (OQ704209, OQ704209), with no intraspecific variation. The sequences obtained for *P. obtusa* (PQ589235, PQ589236) were 100% identical to those of *P. obtusa* isolate from Czech Republic (KJ705089); and highly similar to that of *P. obtusa* isolate accessioned EU003189 and the unidentified *Parasitorhabditis* sp. (AF083028), differing by only 3–4 bp (0.2%). The sequences also differed from short sequence data of *P. obtusa* isolate from Israel (MG865783) by 6 bp (0.6%); but significantly differed from the Taiwan isolate (MH590260) and the unidentified *Parasitorhabditis* spp. sequences (MH590259 and MH590261) by 31–33 bp (3.9–4.2%).

Amplification of the 28S rRNA gene yielded single amplicons of ca 600 bp. The three D2-D3 sequences from *Parasitorhabditis paraterebrana* n. sp. (PQ589220-PQ589222) showed no intraspecific variation. Similar to the 18S rRNA gene tree, the more informative 28S rRNA gene tree also showed close phylogenetic relationships among the available sequences of the genus, and all sequences of *Parasitorhabditis* are grouped in a well-supported clade (PP = 100%). Sequences of *Parasitorhabditis paraterebrana* n. sp. differed from *P. terebrana* sequences (OQ291289, OQ291290 and PQ589223) by 13 bp (2.4%); and from those of the closely similar species *P. obtusa* isolates (MF288651, MG865784, including the new sequences PQ589224, PQ589225), and two other more phylogenetically distant *P. obtusa* isolates (EF990724 and KM245037) by 19 bp (3.2–3.8%) and 25 bp (4.2–4.7%), respectively. The newly obtained sequences of *P. terebrana* (PQ589223) differed from the previously submitted sequences of *P. terebrana* (OQ291289, OQ291290) by 0–1 bp, and the obtained sequences of *P. obtusa* (PQ589224, PQ589225) differed from those of *P. obtusa* isolates (MF288651, MG865784, EF990724 and KM245037) by 7 bp (1.4%).

The amplified partial *COI* gene yielded single amplicons of ca 650 bp. There were only two *COI* sequences of *Parasitorhabditis* available in the GenBank database at the time of analysis (i.e., *P. terebrana* OQ281742 and OQ281743). The newly obtained sequence of *P. terebrana* (PQ585735) was 100% identical to the two aforementioned, available *P. terebrana* sequences. The new sequences of *P. paraterebrana* n. sp. (PQ585733, PQ585734) showed no intraspecific variation and differed from those of *P. terebrana* (OQ281742, OQ281743 and PQ585735) by 95–99 bp (15.4–15.4%). *Parasitorhabditis paraterebrana* n. sp. sequences (PQ585733, PQ585734) also differed from the newly obtained sequences of *P. obtusa* (PQ585736, PQ585737) by 86–93 bp (13.8–14.2%). The two sequences of *P. obtusa* (PQ585736, PQ585737) showed an intraspecific variation of 2 bp. In the reconstructed *COI* gene phylogeny, the three species were closely related and formed a well-supported subclade (PP=100) among other rhabditid species. The amplified partial ITS rRNA gene yielded single amplicons of ca 650–850 bp. Similar to the partial *COI* gene, there were only a few ITS rRNA gene sequences of *Parasitorhabditis* available in the GenBank database; all from one species (i.e., *P. terebrana* OQ305560-OQ305564). No intraspecific variation was recorded in the new sequences of *P. paraterebrana* n. sp. (PQ589226-PQ589228), differing from those of *P. terebrana* (OQ305560-OQ305564 and PQ589229) and *P. obtusa* (PQ589230, PQ589231) by 79–82 bp (12.6–12.9%) and 93–114 bp (14.1–16.2%), respectively. The newly obtained sequence data of *P. terebrana* (PQ589229) was 100% identical to the available *P. terebrana* sequences (OQ305560-OQ305564).

**Figure 5:**
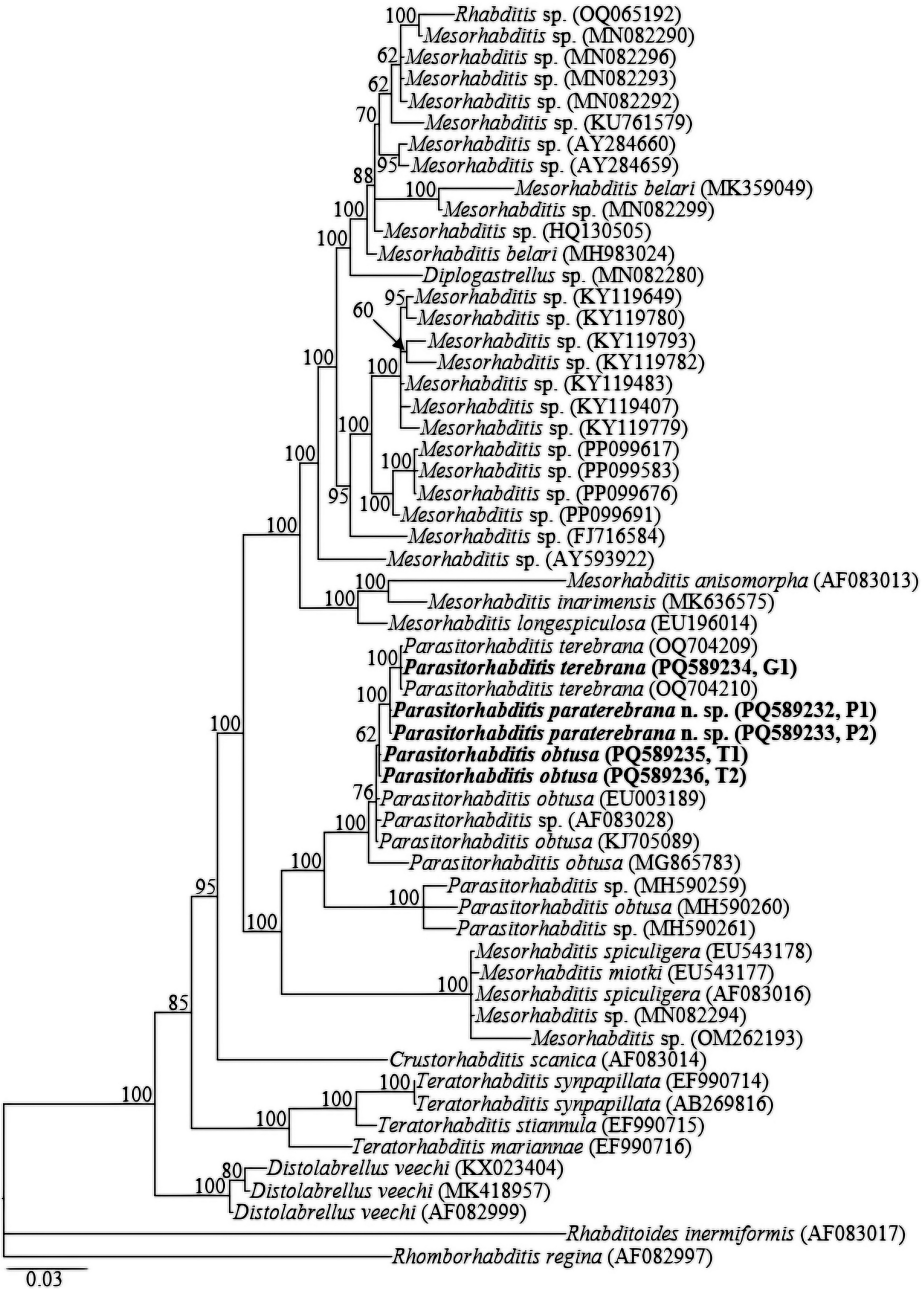
Bayesian tree inferred under the GTR + I + G model from 18S rRNA gene sequences of Rhabditina Chitwood, 1933 species. Posterior probability values exceeding 50 % are given on appropriate clades. The studied population is indicated in bold text. Outgroups: *Rhabditoides inermiformis* (Osche, 1952) Dougherty, 1955 and *Rhomborhabditis regina* (Schulte and Poinar, 1991) Sudhaus, 2011.

**Figure 6:**
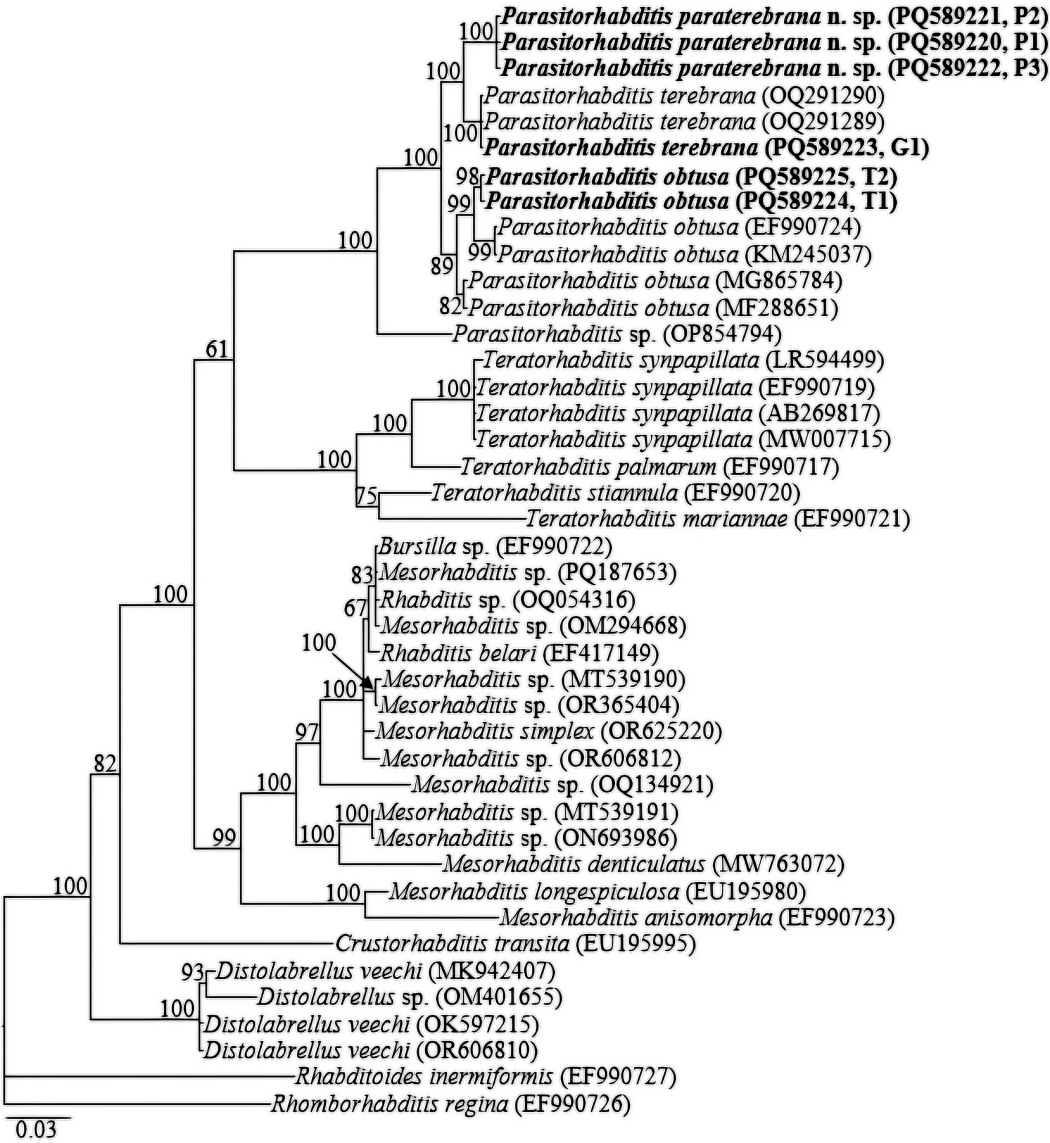
Bayesian tree inferred under the GTR + I + G model from LSU D2-D3 partial sequences of Rhabditina Chitwood, 1933 species. Posterior probability values exceeding 50% are given on appropriate clades. The studied population is indicated in bold text. Outgroups: *Rhabditoides inermiformis* (Osche, 1952) Dougherty, 1955 and *Rhomborhabditis regina* (Schulte and Poinar, 1991) Sudhaus, 2011.

**Figure 7:**
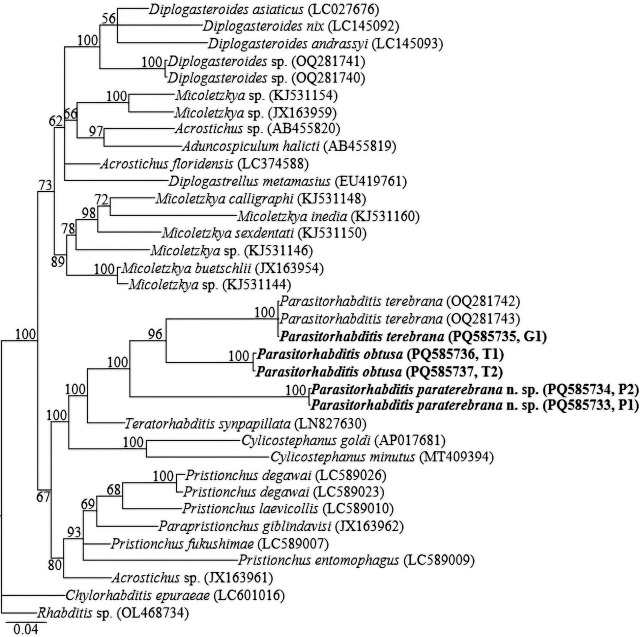
Bayesian tree inferred under the GTR + I + G model from *COI* partial sequences of Rhabditina Chitwood, 1933 species. Posterior probability values exceeding 50% are given on appropriate clades. The studied population is indicated in bold text. Outgroups: *Chylorhabditis epuraeae* Kanzaki, Hamaguchi and Takeuchi-Kaneko, 2021, and *Rhabditis* Dujardin, 1844 species.

Fifty-eight 18S rRNA, forty-two 28S rRNA, and thirty-six *COI* gene sequences from various member species of Rhabditina Chitwood, 1933, inclusive of the newly obtained sequences and outgroups, constituted the sequence dataset for phylogenetic analyses. Phylogenetic relationships, as inferred from Bayesian analysis of the dataset with GTR + I + G substitution model, are shown in Figures 5–7.

## Discussion

Rhabditids, a saprophagous group of nematodes, have always been regarded as a difficult group due to the profusion of highly similar species (Sudhaus and Fitch, 2001). The genus *Parasitorhabditis* is known to be rather uniform biologically and ecologically, with all its species generally known to form parasitic or phoretic relationships with bark beetles, generally being benign to their hosts (Chapman, 1964; Poinar, 1975; Tomalak et al., 1989; Carta et al., 2010). Members of the genus are fairly recognizable. However, species delimitation within the genus shows only slight morphological differences (Sudhaus and Fitch, 2001), and identification of species within the genus is very challenging due to phenotypic plasticity in the key diagnostic characters (Sudhaus and Fitch, 2001). This is exacerbated by the possible existence of cryptic species within the genus. Early taxonomic studies by Rühm (1956, 1960) attempted to categorize at least four species groups within the genus, including the *Ateri*, *Chalcographi*, *Obtusa*, and the *Autographi* groups based on various characters. But no unequivocal characters support the relatedness of the species in each group, and species boundaries are still not well defined. Currently, about 43 species comprise the genus (Andrássy, 1984; Sudhaus, 2011), and since the compendium of Carta et al. (2010), no other new species have been described.

Cryptic species are a common occurrence among various nematode groups including plant-parasitic nematodes, and free-living marine and soil dwelling nematodes; and a number of cryptic species have been described, especially based on genetic divergence among populations with no or slight morphological differences (Palomares-Rius et al., 2014; Olson et al., 2017; Daly et al., 2021; Guden et al., 2024). *Parasitorhabditis paraterebrana* n. sp. is one such species. Here, inferences from the analysis of the more informative D2-D3 expansion segment of 28S rRNA gene, the partial ITS rRNA gene and the partial *COI* gene sequences suggest that *P. paraterebrana* n. sp. is genetically distinct from *P. terebrana* and the few other available *Parasitorhabditis* gene sequences. These distinct genetic lineages of two close morphological species suggest that *P. paraterebrana* n. sp. is a sister species to *P. terebrana* and probably represent a recent species divergence. *Parasitorhabditis* thus appears to be a speciose genus, with the possible existence of more cryptic species. Also, being a taxonomically confounded genus, especially with insufficient morphometric data published in some original descriptions, the taxonomic position of some species is still ambiguous and may require taxonomic resolution using molecular data. For instance, Andrássy (1983) listed *P. masseyi* Sudhaus, 1976 as a junior synonym to *P. subelongati* Slobodianiuk, 1973; *P. ipsophila* Lieutier and Laumond, 1978 to *P. thornei* Sudhaus, 1976; and *P. crypturgophila* Rühm, 1956 to *P. opaci* Rühm, 1956; including listing *P. cembraei* (Fuchs, 1937) Skrjabin, Shikhobalova, Sobolev, Paramonov, and Sudarikov, 1954, *P. crenati* (Fuchs, 1937) Rühm, 1956, *P. montani* (Fuchs, 1915) Sudhaus, 1976 and *P. pini* Lazarevskaya, 1962 as *species inquirendae.* However, all these species have since been reinstated (listed) as valid species of the genus (see Carta et al. (2010) and Nemys (2024)).

It is therefore clear that microscopical data alone cannot provide the adequate diagnostic resolution required to consistently delineate species within this genus. As demonstrated by other nematode taxonomic studies (Subbotin, 2015; Mwamula et al., 2020, 2022), integrated identification that considers both morphological characters and molecular phylogenetic inferences provides a better-supported approach in delineating cryptic species and conspecific populations that display deceptive morphological variations. Unfortunately, DNA sequence data of many of these species are still unavailable. This is partly due to the fact that species of this group have not been given the necessary attention, since more emphasis is usually attributed to the destructive nematode species of agricultural importance. It is therefore imperative that DNA sequences from populations recovered from type localities be obtained as this will resolve the taxonomic position of the various species of the genus.

## References

[j_jofnem-2025-0027_ref_001] Andrássy I. (1983). A taxonomic review of the suborder Rhabditina (Nematoda: Secernentia).

[j_jofnem-2025-0027_ref_002] Andrássy I. (1984). Klasse nematoda.

[j_jofnem-2025-0027_ref_003] Baermann G. (1917). Eine einfache methode zur auffindung von ankylostomum (nematoden) larven in erdproben. Geneeskundig Tijdschrift voor Nederlandsch-Indie.

[j_jofnem-2025-0027_ref_004] Bhat A. H., Srivastava S., Rana A., Chaubey A. K., Machado R. A., Abolafia J. (2020). Morphological, morphometrical, and molecular characterization of *Metarhabditis amsactae* (Ali, Pervez, Andrabi, Sharma and Verma, 2011) Sudhaus, 2011 (Rhabditida, Rhabditidae) from India and proposal of *Metarhabditis longicaudata* as a junior synonym of *M. amsactae*. Journal of Nematology.

[j_jofnem-2025-0027_ref_005] Carta L. K., Bauchan G., Hsu C. Y., Yuceer C. (2010). Description of *Parasitorhabditis frontali* n. sp. (Nemata: Rhabditida) from *Dendroctonus frontalis* Zimmermann (Coleoptera: Scolytidae). Journal of Nematology.

[j_jofnem-2025-0027_ref_006] Chapman J. A. (1964). Nematode infestation and sex difference in response to log odours, in the cerambycid beetle, *Leptura obliterata* (Haldeman). Bi-Monthly Progress Report Forestry, Canada.

[j_jofnem-2025-0027_ref_007] Chitwood B. G. (1933). On some nematodes of the superfamily Rhabditoidea and their status as parasites of reptiles and amphibians. Journal of the Washington Academy of Sciences.

[j_jofnem-2025-0027_ref_008] Chitwood B. G., Chitwood M. B. (1950). An introduction to Nematology.

[j_jofnem-2025-0027_ref_009] Curran J., Driver F., Ballard J. W. O., Milner R. J. (1994). Phylogeny of *Metarhizium*: Analysis of ribosomal DNA sequence data. Mycological Research.

[j_jofnem-2025-0027_ref_010] Daly A. J., De Meester N., Baetens J. M., Moens T., De Baets B. (2021). Untangling the mechanisms of cryptic species coexistence in a nematode community through individual-based modelling. Oikos.

[j_jofnem-2025-0027_ref_011] De Grisse A. T. (1969). Redescription ou modifications de quelques techniques utilisées dans l’étude des nématodes phytoparasitaires. Mededelingen Rijksfaculteit Landbouwwetenschappen Gent.

[j_jofnem-2025-0027_ref_012] De Ley P., Felix M. A., Frisse L., Nadler S., Sternberg P., Thomas W. K. (1999). Molecular and morphological characterisation of two reproductively isolated species with mirror-image anatomy (Nematoda: Cephalobidae). Nematology.

[j_jofnem-2025-0027_ref_013] Dougherty E. C. (1955). The genera and species of the subfamily Rhabditinae Micoletzky, 1922 (Nematoda): A nomenclatorial analysis, including an addendum on the composition of the family Rhabditidae Örley, 1880. Journal of Helminthology.

[j_jofnem-2025-0027_ref_014] Dujardin F. (1844. (“1845”)). Histoire naturelle des helminthes ou vers intestinaux, xvi + 654 + 15 pp., pls. 1–12.

[j_jofnem-2025-0027_ref_015] Fuchs G. (1915). Die naturgeschichte der nematoden und einiger anderer Parasite. 1. des Ips typographus L. 2. des Hylobius abietis L.. Zool. Jahrb. (Syst.).

[j_jofnem-2025-0027_ref_016] Fuchs G. (1937). Neue parasitische und halbparasitische Nematoden bei Borkenkäfern und einige andere Nematoden. 1. Teil. Zoologische Jahrbücher (Systematik).

[j_jofnem-2025-0027_ref_017] Guden R. M., Derycke S., Moens T. (2024). Resource diversity mitigates the effects of intraspecific competition in co-occurring cryptic nematode species. Frontiers in Marine Science.

[j_jofnem-2025-0027_ref_018] Grucmanová Š., Holuša J. (2013). Nematodes associated with bark beetles, with focus on the genus *Ips* (Coleoptera: Scolytinae) in Central Europe. Acta Zoologica Bulgarica.

[j_jofnem-2025-0027_ref_019] Holterman M., van der Wurff A., van den Elsen S., van Megen H., Bongers T., Holovachov O., Bakker J., Helder J. (2006). Phylum-wide analysis of SSUrDNA reveals deep phylogenetic relationships among nematodes and accelerated evolution toward crown clades. Molecular Biology and Evolution.

[j_jofnem-2025-0027_ref_020] Iwahori H., Kanzaki N., Futai K. (2000). A simple, polymerase chain reaction-restriction fragment length polymorphism-aided diagnosis method for pine wilt disease. Forest Pathology.

[j_jofnem-2025-0027_ref_021] Kanzaki N., Futai K. (2002). A PCR primer set for determination of phylogenetic relationships of *Bursaphelenchus* species within the xylophilus group. Nematology.

[j_jofnem-2025-0027_ref_022] Kanzaki N., Hamaguchi K., Takeuchi-Kaneko Y. (2021). *Chylorhabditis epuraeae* n. gen., n. sp. (Rhabditida: Rhabditidae) isolated from *Epuraea* (*Haptoncus*) *ocularis* Fairmaire collected from sap on the bark of Ulmus parvifolia Jacq. in Kyoto, Japan. Nematology.

[j_jofnem-2025-0027_ref_023] Kanzaki N., Sakamoto H., Maehara N. (2016). *Diplogasteroides nix* n. sp.(Nematoda: Diplogastridae), a cryptic species related to *D. andrassyi*, isolated from *Monochamus urussovii* (Coleoptera: Cerambycidae) from Hokkaido, Japan, with remarks on body surface structures. Nematology.

[j_jofnem-2025-0027_ref_024] Kanzaki N., Tanaka R., Hirooka Y., Maehara N. (2013). Description of *Diplogasteroides andrassyi* sp. n. (Rhabditida, Diplogastridae), associated with *Monochamus grandis* and Pinaceae trees in Japan. Journal of Nematode Morphology and Systematics.

[j_jofnem-2025-0027_ref_025] Kanzaki N., Woodruff G. C., Akiba M., Maehara N. (2015). *Diplogasteroides asiaticus* n. sp. is associated with *Monochamus alternatus* in Japan. Journal of Nematology.

[j_jofnem-2025-0027_ref_026] Lazarevskaya S. L. (1962). New species of nematode of Pine weevil (Pissodes pini L.). Tr. Gel’mintol. Lab. (Akademija Nauk SSSR).

[j_jofnem-2025-0027_ref_027] Lieutier F., Laumond C. (1978). Nématodes parasites et associés à *Ips sexdentatus* et *Ips typographus* (Coleoptera, Scolytidae) en région parisienne. Nematologica.

[j_jofnem-2025-0027_ref_028] Massey C. L. (1956). Nematode parasites and associates of the *Engelmann spruce* beetle (Dendroctonus engelmann Hook.). Proceedings of the Helminthological Society of Washington.

[j_jofnem-2025-0027_ref_029] Massey C. L. (1960). Nematode parasites and associates of the California five-spined engraver, *Ips confusus* (Lec.). Proceedings of the Helminthological Society of Washington.

[j_jofnem-2025-0027_ref_030] Massey C. L. (1974). Biology and taxonomy of nematode parasites and associates of bark beetles in the United States No. 446.

[j_jofnem-2025-0027_ref_031] Mwamula A. O., Kwon O. G., Kwon C., Kim Y. S., Kim Y. H., Lee D. W. (2024). A revision of the phylogeny of *Helicotylenchus* Steiner, 1945 (Tylenchida: Hoplolaimidae) as inferred from ribosomal and mitochondrial DNA. The Plant Pathology Journal.

[j_jofnem-2025-0027_ref_032] Mwamula A. O., Lee G., Kim Y. H., Kim Y. H., Lee K. S., Lee D. W. (2020). Molecular phylogeny of several species of Hoplolaimina (Nematoda: Tylenchida) associated with turfgrass in Korea, with comments on their morphology. Nematology.

[j_jofnem-2025-0027_ref_033] Mwamula A. O., Lee S. M., Jung Y. H., Lee H. W., Kim Y. S., Kim Y. H., Lee D. W. (2023). Morphological and molecular characterization of *Diplogasteroides* sp., a cryptic population of the Haslacheri Group (Diplogastridae), and Parasitorhabditis terebrana (Rhabditidae) from Korea. Journal of Nematology.

[j_jofnem-2025-0027_ref_034] Mwamula A. O., Lim T. H., Kim Y., Lee H. W., Kim Y. H., Lee D. W. (2022). Morphological plasticity in the rice root nematode, *Hirschmanniella oryzae* (van Breda de Haan, 1902) Luc & Goodey, 1964 from Korea, with inferences from its ribosomal and mitochondrial DNA. European Journal of Plant Pathology.

[j_jofnem-2025-0027_ref_035] Nemys (2024). Nemys: World database of nematodes. Parasitorhabditis Fuchs, 1937.

[j_jofnem-2025-0027_ref_036] Nunn G. B. (1992). Nematode molecular evolution.

[j_jofnem-2025-0027_ref_037] Olson M., Harris T., Higgins R., Mullin P., Powers K., Olson S., Powers T. O. (2017). Species delimitation and description of *Mesocriconema nebraskense* n. sp. (Nematoda: Criconematidae), a morphologically cryptic, parthenogenetic species from North American grasslands. Journal of Nematology.

[j_jofnem-2025-0027_ref_038] Örley L. (1880). Az Anguillulidák magánrajza. (Monographie der Anguilluliden). Természetrajzi Füzetek.

[j_jofnem-2025-0027_ref_039] Osche G. (1952). Systematik und phylogenie der gattung *Rhabditis* (Nematoda). Zoologische Jahrbücher (Systematik).

[j_jofnem-2025-0027_ref_040] Palomares-Rius J. E., Cantalapiedra-Navarrete C., Castillo P. (2014). Cryptic species in plant-parasitic nematodes. Nematology.

[j_jofnem-2025-0027_ref_041] Poinar G. O. (1975). Entomogenous nematodes: A manual and host list of insect-nematode associations.

[j_jofnem-2025-0027_ref_042] Ronquist F., Teslenko M., Van Der Mark P., Ayres D. L., Darling A., Höhna S., Larget B., Liu L., Suchard M. A., Huelsenbeck J. P. (2012). MrBayes 3.2: Efficient Bayesian phylogenetic inference and model choice across a large model space. Systematic Biology.

[j_jofnem-2025-0027_ref_043] Rühm W. (1956). Die nematoden der Ipiden. Parasitologische Schriftenreihe.

[j_jofnem-2025-0027_ref_044] Rühm W. (1960). Ein Beitrag zur Nomenklatur und Systematik einiger mit Scolytiden vergesellschafteter Nematodenarten. Zoologischer Anzeiger.

[j_jofnem-2025-0027_ref_045] Schulte F., Poinar G. J. (1991). Description of *Rhabditis* (*Rhabditoides*) *regina* n. sp. (Nematoda: Rhabditidae) from the body cavity of beetle larvae in Guatemala. Revue Nématol.

[j_jofnem-2025-0027_ref_046] Seinhorst J. W. (1959). A rapid method for the transfer of nematodes from fixative to anhydrous glycerin. Nematologica.

[j_jofnem-2025-0027_ref_047] Skrjabin K. J., Shikhobalova N. P., Sobolev A. A., Paramonov A. A., Sudarikov V. E. (1954). Kamalanaty, rabditaty, tilenchaty, trikhotsefaliaty, dioktofimaty i raspredelenie paraziticheskikh nematod po khozyaevam. Opredelitel’paraziticheskikh nematod.

[j_jofnem-2025-0027_ref_048] Slobodianiuc O. V. (1973). *Parasitorhabditis subelongati* (Parasitorhabditinae, Rhabditida), a new species of nematodes from Ips subelongatus. Zoologichesky Zhurnal.

[j_jofnem-2025-0027_ref_049] Subbotin S. A. (2015). *Heterodera sturhani* sp. n. from China, a new species of the *Heterodera avenae* species complex (Tylenchida: Heteroderidae). Russian Journal of Nematology.

[j_jofnem-2025-0027_ref_050] Sudhaus W. (1974). Zur systematik, verbreitung, oekologie und biologie neuer und wenig bekannter rhabditiden (Nematoda) 2. teil. Zoologische jahrbucher. Abteilung fur Systematik, Okologie und Geographie der Tiere.

[j_jofnem-2025-0027_ref_051] Sudhaus W. (1976). Nomenklatorische bemerkungen uber arten und gattungen der unterfamilie rhabditinae sensu lato (Rhabditidae, Nematoda). Nematologica.

[j_jofnem-2025-0027_ref_052] Sudhaus W. (2011). Phylogenetic systematisation and catalogue of paraphyletic “Rhabditidae” (Secernentea, Nematoda). Journal of Nematode Morphology and Systematics.

[j_jofnem-2025-0027_ref_053] Sudhaus W., Fitch D. (2001). Comparative studies on the phylogeny and systematics of the Rhabditidae (Nematoda). Journal of Nematology.

[j_jofnem-2025-0027_ref_054] Swofford D. L. (2003). PAUP*: Phylogenetic analysis using parsimony and other methods. Version 4.0a165.

[j_jofnem-2025-0027_ref_055] Thompson J. D., Gibson T. J., Plewniak F., Jeanmougin F., Higgins D. G. (1997). The CLUSTAL_X windows interface: Flexible strategies for multiple sequence alignment aided by quality analysis tools. Nucleic Acids Research.

[j_jofnem-2025-0027_ref_056] Tomalak M., Welch H. E., Galloway T. D. (1989). Parasitism of *Parasitorhabditis obtusa* and *P. autographi* (Nematoda: Rhabditidae) in the digestive tract of their bark beetle (Coleoptera: Scolytidae) hosts. Journal of Invertebrate Pathology.

[j_jofnem-2025-0027_ref_057] Valizadeh A., Goldasteh S., Rafiei-Karahroodi Z., Pedram M. (2017). First record of the genus *Parasitorhabditis* Fuchs, 1937 (Rhabditida, Nematoda) from Iran with notes on morphological and molecular characters of the Iranian population of *P. obtusa* (Fuchs, 1915) Chitwood & Chitwood, 1950. Zootaxa.

[j_jofnem-2025-0027_ref_058] Vrain T. C., Wakarchuk D. A., Levesque A. C., Hamilton R. I. (1992). Intraspecific rDNA restriction fragment length polymorphism in the *Xiphinema americanum* group. Fundamental and Applied Nematology.

